# Substitute for Tobacco

**Published:** 1881-05

**Authors:** Frank B. Bullard

**Affiliations:** Chestnut, Illinois


### SUBSTITUTE FOR TOBACCO.

Ed. Med. and Surg. Reporter.

In looking over the back volumes of your Journal, I noticed in January 3d 1880, number, a question by a Dr. B. L. L., of Illinois, asking, "Is there any harmless substitute, or drug, which will induce a distaste for tobacco?" And in reply, would say, that about eighteen months ago I was attending a young man who had useed tobacco to such an extent that it was affecting his nervous system, and having used it for a number of years he found it difficult to do without something to chew, as he stated it, when I suggested that he try common chewing gum for a while, until his system could rebuild itself again. After having used the common gum (that is, no particular make), for two or three weeks, he has had no desire for tobacco, and has not taken a chew since he first began using the gum.

In the last six or eight months I have recommended it to several tobacco chewers (one of thirty years' standing), with like success, and I begin to think it is a cure that at least only costs a trifle to try it, and is not an unpleasant experiment. I am sincere when I say to the medical profession that it is the nearest a harmless, and I think, a successful, antidote for tobacco of anything that we have ever used.

It was discovered by the merest accident, and by giving it to the profession I am desirous of hearing of their failures and successes elsewhere with it. FRANK B. BULLARD, M. D.

*Chestnut, Illinois, January 14th,* 1881.

